# Performance evaluation of Zr(CUR)/NiCo_2_S_4_/CuCo_2_S_4_ and Zr(CUR)/CuCo_2_S_4_/Ag_2_S composites for photocatalytic degradation of the methyl parathion pesticide using a spiral-shaped photocatalytic reactor†

**DOI:** 10.1039/d2ra06277a

**Published:** 2022-10-14

**Authors:** Hamideh Zolfaghari, Fakhri Yousefi, Mehrorang Ghaedi, Soleiman Mosleh

**Affiliations:** Chemistry Department, Yasouj University Yasouj 75918-74831 Iran fyousefi@yu.ac.ir m_ghaedi@yu.ac.ir; Polymer Engineering Department, Faculty of Gas and Petroleum, Yasouj University Gachsaran 75813-56001 Iran

## Abstract

Zr(CUR)/NiCo_2_S_4_/CuCo_2_S_4_ and Zr(CUR)/CuCo_2_S_4_/Ag_2_S ternary composites were synthesized as efficient photocatalysts, and well characterized through XRD, FTIR, DRS, FE-SEM, EDS, and EDS mapping techniques. The potential of a spiral-shaped photocatalytic reactor was evaluated for degradation of the methyl parathion (MP) pesticide using synthesized photocatalysts under visible light irradiation. Computational fluid dynamics (CFD) was applied for analysis of the hydrodynamics behaviour and mass transport occurring inside the reactor. The experiments were performed based on a developed CCD-RSM model, while the desirability function (DF) was used for optimization of the process. Findings showed that the highest MP degradation percentage was 98.70% at optimal operating values including 20 mg L^−1^, 0.60 g L^−1^, 8 and 40 min for MP concentration, catalyst dosage, pH, and reaction time, respectively. This study clearly demonstrated that high degradation efficiency can be achieved using a spiral-shaped photocatalytic reactor rather than a traditional annular reactor at same conditions. The increase in reaction rate is related to the higher average turbulence kinetic energy in the spiral-shaped reactor over the traditional reactor, which results in the increased diffusivity and improves the mass and momentum transfer.

## Abstract 2

Fabrication of Zr(CUR)/NiCo_2_S_4_/CuCo_2_S_4_ and Zr(CUR)/CuCo_2_S_4_/Ag_2_S composites as an efficient photocatalyst. Examination of the potential of a spiral-shaped photocatalytic reactor for degradation of the methyl parathion pesticide.

## Graphical abstract

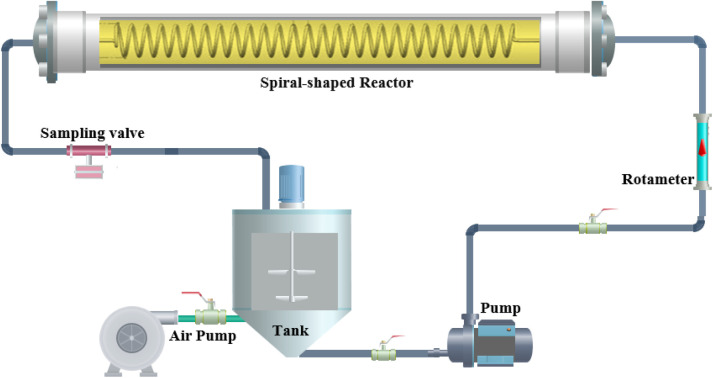

## 1. Introduction

The presence of recalcitrant organic contaminants in wastewaters has caused serious environmental concern.^1,2^ In this regard, the photocatalytic degradation as one of the advance oxidation processes is proposed for the treatment of such recalcitrant organic pollutants in wastewaters.^3,4^ The performance of the photocatalysts is still unsatisfactorily related to high recombination efficiency of electron–hole pairs and photo-corrosion.^5^ In order to utilize the photocatalyst degradation process for the treatment of the contaminants, various semiconductors have been synthesized. Nonetheless, some disadvantages including fast recombination of photo-generated electron–hole pairs, lower light response, poor photo-stability, and low efficiency in solar light harvesting restrict their practical application.^6^ There are different strategies to overcome the drawbacks above, such as the construction of composite structures, treatment of precursors or products, change of synthetic conditions, doping, and exfoliation. Among them, the construction of composite structures is well-known as an effective modification approach for boosting photocatalytic performance. CuCo_2_S_4_ is a typical thiospinel compound that is used in many fields such as the preparation of solar cells,^7^ construction of supercapacitors,^8^ and production of lithium-ion batteries.^9^ Several properties of CuCo_2_S_4_ including the electronic band structure, thermal stability, and controllable morphology make it a unique photocatalyst.^10^ However, the CuCo_2_S_4_ photocatalyst suffers from some restrictions such as undersized specific surface area, poor visible-light absorption, and low carrier mobility.^11^ The strategy of construction of composite structures could help to improve the visible-light absorption and to enhance the surface area of CuCo_2_S_4_.^12^ The binary metallic sulfides are useful candidates for the construction of composites to enhance photocatalytic performance. Ni–Co sulfide and phosphides have been found to exhibit strong electrochemical performance owing to their small bandgap energy and high conductivity.^13^ Generally, binary Ni–Co sulfides leads to rich redox reactions and higher electrochemical activity than single metal sulfides such as NiS and CoS_2_, because of different valence states of Ni and Co.^14^ Hence, in this work NiCo_2_S_4_ is selected as an excellent material for coupling to CuCo_2_S_4_. In recent years, different types of binary and ternary composites containing NiCo_2_S_4_ were proposed for photocatalytic degradation purposes. NiCo_2_S_4_/ZnIn_2_S_4_,^14^ NiCo_2_S_4_/ZnS/CdS,^15^ and ZnO/NiCo_2_S_4_ QDs-OVs^16^ are examples of constructed materials to degrade organic pollutants. Furthermore, Ag_2_S as a n-type semiconductor with unique structure and excellent optical properties was chosen for coupling to NiCo_2_S_4_ and CuCo_2_S_4_ to preparing high-performance photocatalysts. The presence of Ag_2_S in the composite could increase the light absorbance and accelerate the separation of photogenerated charge.

One of the big challenges in the photocatalytic degradation process is the low quantum efficiency of conventional semiconductors because the photogenerated electrons and holes recombine easily. To remove this challenge, elemental doping is an efficient technique, since this approach could broaden the range of the visible light response and limits the recombination of electron–hole pairs, enhancing photocatalytic process performance.^17^ Among the various metals, zirconium (Zr) being a biocompatible material is an acceptable candidate as a dopant,^18^ while Zr^4+^ ions with smaller size and lower valence could generate positive holes with lattice defects (oxygen vacancies).^19^ Zr can cause lattice defects and surface modification, changing its photocatalytic performance.^20^ Zirconium doping suppresses electron–hole recombination and increases the specific surface area that is most important for the enhanced photocatalytic activity of various metal oxides such as TiO_2_,^21^ SiO_2_,^22^ CeO_2_,^23^ CrO_2_,^24^*etc.*

Curcumin acts as a photosensitizer owing to an appropriate HOMO-LUMO gap in the visible range.^25^ Curcumin can be coupled with wide band gap photocatalysts to produce visible-light-driven photocatalyst.^26^ It gets photo-excited when the material is illuminated with visible light. The excited electron is transferred from the LUMO of curcumin to the wide bandgap semiconductor CB. In fact, curcumin with its intensive yellow color is a suitable candidate to shift the photocatalytic response of the sample to the visible region and prevent electron–hole recombination.^27^ The presence of curcumin in the structure of Zr(CUR)/NiCo_2_S_4_/CuCo_2_S_4_ and Zr(CUR)/CuCo_2_S_4_/Ag_2_S composites is accompanied by electron transfer from excited energy level of curcumin which increases the reactive oxygen species [O_2_˙] and prevents electron–hole recombination. Generally, the superior photocatalytic reactivity of the prepared Zr(CUR)-based photocatalysts is corresponded to the reduction in the band gap energy and to the facility of electron transfer from curcumin energy level which increases the concentration of reactive oxygen superoxide radicals which in turn preventing the electron–hole recombination.

Accordingly, Zr(CUR)/NiCo_2_S_4_/CuCo_2_S_4_ and Zr(CUR)/CuCO_2_S_4_/Ag_2_S composites were selected as novel candidates to production of superior photocatalysts. One of the main advantage of this work is utilizing the Zr(CUR) for the construction of the ternary composites. The superior photocatalytic reactivity of the prepared Zr(CUR)-based photocatalysts is corresponded to the reduction in the band gap energy and to the facility of electron transfer from curcumin energy level which increases the concentration of reactive oxygen superoxide radicals which in turn preventing the electron–hole recombination.

Despite different performed researches, the design of an efficient photocatalytic reactor still remained as a major challenge owing to the mass transfer restriction and maldistribution of light in the reactor domain.^28,29^ The photocatalytic reactors are mainly classified into slurry reactors and immobilized reactors according to the catalyst status.^30^ In this research, an efficient slurry photocatalytic reactor called spiral-shaped photocatalytic reactor has been developed to the degradation of the methyl parathion (MP) as a recalcitrant pesticide under visible light irradiation. Zr(CUR)/NiCo_2_S_4_/CuCo_2_S_4_ and Zr(CUR)/CuCo_2_S_4_/Ag_2_S composites were synthesized as the photocatalyst. In this reactor, an excellent light distribution is created in the domain, while a great contact surface area is provided between the contaminant and the photocatalyst. The wide reaction region is another advantage of this reactor, which leads to supreme degradation performance. Furthermore, the spiral-shaped reactor has a very compact design, unlike the traditional photocatalytic reactors.^31^ In order to achieve the effects of individual operation parameters and their interactions, the central composite design (CCD) was applied in agreement with the response surface methodology (RSM). The optimization of the MP degradation process was finalized through the desirability function (DF).^32,33^ Furthermore, the experimental results were completed by computational fluid dynamic (CFD) calculations that are validated by the experiments. CFD is a robust tool for the simulation of the photocatalytic process which can accurately predict different physical and chemical behaviours of the species in the reactor domain during the process.^28,34^ Finally, the performance of the designed spiral-shaped reactor was compared with a conventional annular reactor in same conditions. Findings indicated that spiral-shaped reactor is more economic and has higher efficiency. It is expected that the results of this study could be an essential step during the designing and optimization of the photocatalytic reactors, especially for the scale-up process.

## 2. Experiments and CFD simulation

### 2.1. Material

Curcumin (C_21_H_20_O_6_), silver nitrate (AgNO_3_), cobalt(ii) acetate (Co(CH_3_COO)_2_·4H_2_O), nickel(ii) acetate (Ni(CH_3_COO)_2_·4H_2_O), copper acetate (Cu(CH_3_COO)_2_·4H_2_O), thiourea (CH_4_N_2_S), ethylenediamine (C_2_H_4_ (NH_2_)_2_), DMF ((CH_3_)_2_NCH), and ethanol (C_2_H_5_OH) were purchased from Sigma-Aldrich. All the chemicals were used as received without further purification.

### 2.2 Synthesis of the Zr(CUR)

The Zr(CUR) nanoparticles were prepared as follow: curcumin (0.5 g) and zirconium (0.2 g) were dissolved in 50 mL of solvent (4 : 1 v/v ratio) of DMF and ethanol and the mixture was stirred for 2 h. The homogenous solution was exposure to 140 °C for 15.0 h in Teflon-lined autoclave. The product was collected, washed well with ethanol, and dried at 60 °C for 4 h. The synthesized Zr(CUR) solid were stored in a vacuum until uses.

### 2.3. Synthesis of the Zr(CUR)/NiCo_2_S_4_/CuCo_2_S_4_ composite

The Zr(CUR)/NiCo_2_S_4_/CuCo_2_S_4_ composite was prepared by a hydrothermal synthesis method as follows. In a typical procedure, 0.5 g of as-obtained Zr(CUR) was dispersed into 40 mL distilled water and was stirred for 10 min. Then 5 mmol Co(CH_3_COO)_2_·4H_2_O, 1 mmol Ni(CH_3_COO)_2_·4H_2_O and 1 mmol Cu(CH_3_COO)_2_·4H_2_O were dissolved in obtain solution under vigorous stirring for 30 min. Subsequently, 9 mmol CH_4_N_2_S and 4 mL C_2_H_4_ (NH_2_)_2_ were added into the resultant mixture and it was stirred again for 20 min. Finally, the mixture was transferred into a Teflon lined stainless steel autoclave and maintained for 12 h at 200 °C. After naturally cooling down to room temperature, the result precipitate was washed with distilled water and ethanol for three times and dried at 60 °C in the oven for 6 h.

### 2.4. Synthesis of the Zr(CUR)/CuCo_2_S_4_/Ag_2_S composite

The Zr(CUR)/CuCo_2_S_4_/Ag_2_S composite were synthesized by following the similar procedure as mentioned above from their respective precursors. So 0.5 g of Zr(CUR) was dispersed into 20 mL distilled water and was stirred for 10 min. Then 2 mmol Co(CH_3_COO)_2_·4H_2_O, 1 mmol Cu(CH_3_COO)_2_·4H_2_O and 2 mmol AgNO_3_ were dissolved in obtain solution under vigorous stirring for 30 min. To the above mixture, 6 mmol thiourea dissolved in water was added slowly and it was stirred again for 20 min. The resulting reaction mixture was heated at 200 °C in an autoclave for 12 hours. The final product was collected by centrifugation, washed with ethanol and DI water for several times, and dried at 70 °C under vacuum.

### 2.5. Photocatalytic reactor and experiments

**Fig. 1:**
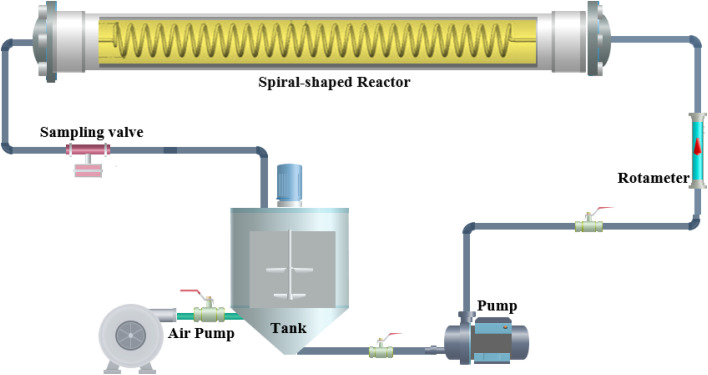
Schematic of the spiral-shaped photocatalytic reactor.

The operation mode of the spiral-shaped photocatalytic reactor is represented in Fig. 1. The reactor was made using a transparent glass pipe with 5 mm inner diameter and 25 cm length. Throughout the time of degradation process, MP solution and photocatalyst particles are mixed in the storage reservoir, while an aeration pump provides the required oxygen for the generation of free oxidant radicals. The uniform irradiance in the reactor domain was supplied using strip LEDs. The characteristics of the used LED including power, luminous intensity, *etc.* were presented in Table S2.† The designed system was shielded by aluminium foil to restrict the ray dispersion. To assessment of the interactions among the operational factors, the MP degradation tests were planned using CCD-RSM. The primary effective factors on the MP degradation process were selected as the independent variables including the photocatalyst loading (*X*_1_, mg L^−1^), pH of solution, MP concentration (*X*_3_, mg L^−1^) and reaction time (*X*_4_, min), while the MP degradation percentage was considered as the desired response (*Y*). The tests planned for the MP degradation process are represented in Table 1, while the obtained results are demonstrated in Table S3.† CCD-RSM provides a mathematical relationship (quadratic model) among the MP degradation efficiency and operational factors as follows:^35^1
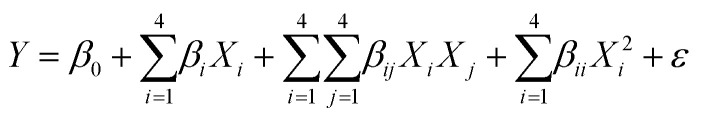
where *Y* is related to MP degradation efficiency; *β*_0_ denotes the constant coefficient; *β*_*i*_ is the linear coefficients; *β*_*ii*_ is corresponded to the quadratic coefficients; *β*_*ij*_ is the interaction coefficients; *X*_*i*_ and *X*_*j*_ are the main operational factors; and *ε* refers to the error.

**Table 1:** The tests planned for the MP degradation process

Factors	Levels				
	Low (−1)	Central (0)	High (+1)	−*α*	+*α*
*X*_1_: catalyst loading (g L^−1^)	0.40	0.60	0.80	0.20	1.0
*X*_2_: pH	4	6	8	2	10
*X*_3_: initial MP concentration (mg L^−1^)	15	20	25	10	30
*X*_4_: irradiation time (min)	20	30	40	10	50

The final extracted quadratic model was verified by a detailed set of statistical evidence along with analysis of variance (ANOVA), including Fisher variation ratio (*F*-value), probability value (*P*-value) and Lack of Fit.

### 2.6. CFD simulation

**Fig. 2:**
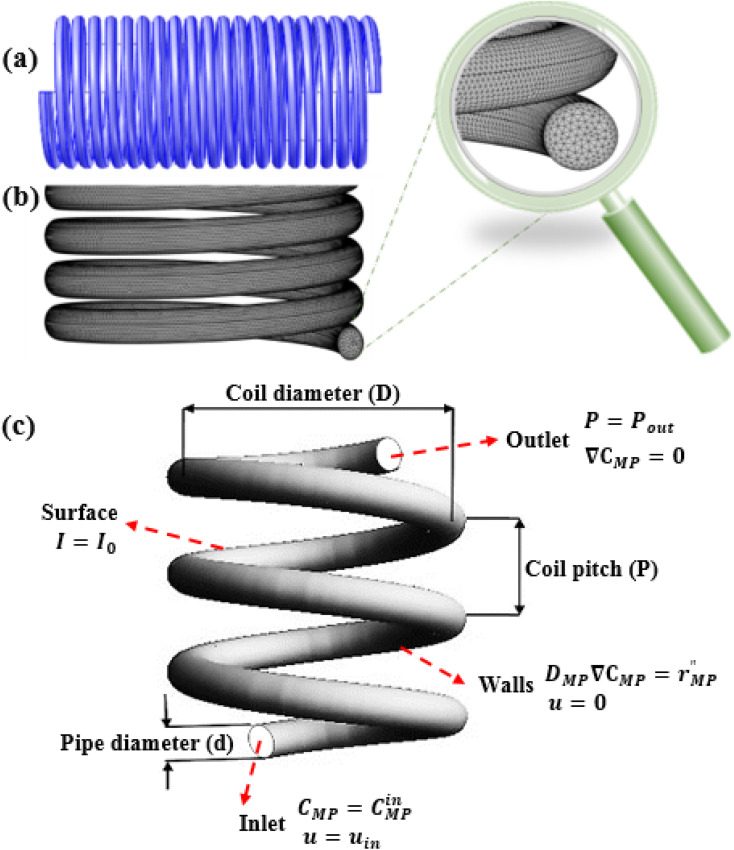
Schematic sketch of the geometry (a), mesh (b), and boundary conditions (c).

CFD simulation was carried out using COMSOL multiphysics software (version 5.4) to design and characterization of the spiral-shaped photocatalytic reactor. The spiral-shaped photocatalytic reactor geometry as well as the mesh generation created by COMSOL are represented in Fig. 2a and b. The reactor consists of glass tube with 5 mm inner diameter and 25 cm length. The hexahedral meshes were applied to discretize the photocatalytic spiral-shaped reactor geometry. The applied grid for the reactor had about 900 000 elements, which were validated to mesh sensitivity and mesh independence study.

The Navier–Stocks and the continuity equations were used to description of the hydrodynamics in the reactor domain based on eqn (2) and (3):^36^2
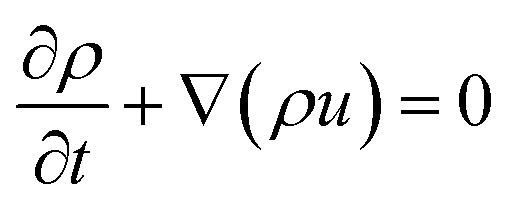
3
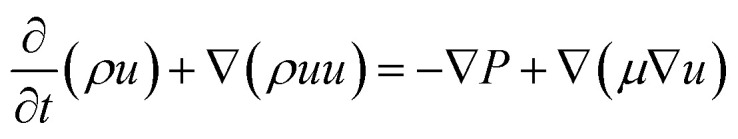
where *u* refers to the velocity vector, *P* denotes the pressure, *ρ* is the MP solution density and *μ* is the viscosity. Only one coil pipe geometry (with three specified main geometrical parameters including the coil pitch (*P*), the pipe diameter (*d*), and the coil diameter (*D*)) was used for the construction of the reactor. The values of the Reynolds (Re) and the Dean number (
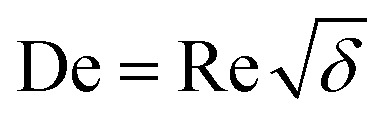
, where 
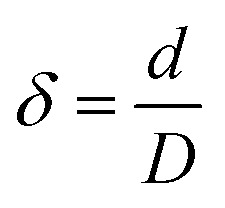
) were 950 and 380, respectively.

The concentration of each chemical species in the control volume was estimated according to the eqn (4):^36,37^4
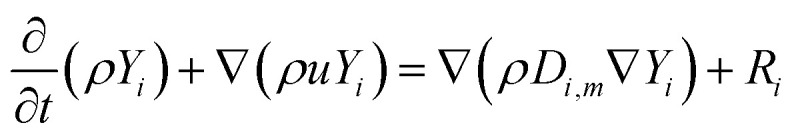
where *Y*_*i*_ denotes the mass fraction, *D*_*i*,*m*_, is related to the mass diffusivity in the MP solution, *R*_*i*_ is the mass source that corresponded to the chemical reactions.

In the photocatalytic reactors, the species react on the photocatalyst surface, whereas the photoreaction rate is governed by the adsorption–desorption equilibrium. The rate of photocatalytic degradation in the slurry photocatalytic reactors depends on the operating conditions including the pH of the solution, temperature, irradiance, oxygen concentration, pollutant concentration, and photocatalyst loading. Hence, the reaction rate is given by:^38^

5 −*r* = *k*(*f*[P])

where,

6 *k* = *f*(pH·*T*·*E*·[O_2_]·*W*_cat._)

The *f*[P] is a function of the pollutant concentration and is usually of the first order or Langmuir–Hinshelwood form.

By consideration of the value of the *L*^*a*^ and its relation to light intensity, the reaction rate can be modified as follows:

7 −*r* = *k*(*L*^*a*^)^*m*^(*f*[P])

When a large amount of photocatalyst is loaded, the reaction rate usually decreases owing to a higher electron–hole recombination rate. Consequently, the volumetric rate of recombination directly corresponds to the photocatalyst loading (*W*_cat_.). Therefore, the final form of the kinetic equation yields as follows:^38^

8 −*r* = *k*_1_(*f*[P])[*L*^*a*^ − *k*_2_*W*_cat._]

In this equation, the second term in between the square brackets on the right hand side corresponds to the increasing rate of electron–hole recombination at increasing catalyst loading.

It is required to solve the radiative transport equation (RTE) to gain the irradiation flux distribution emanating from the source. When the slurry system is used, for a monochromatic ray of light or wavelength band (interval) of intensity (*I*) and wavelength (λ or interval Δ*λ*) traveling in the direction *s* and solid angle (*Ω*) through an absorbing and scattering medium, the RTE for an elemental distance (d*s*) is solved using following equation:^39^9
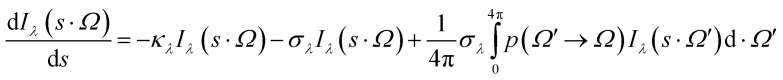


The first term on the right hand side of this equation is related to the absorbed radiation, while the second term denotes the out-scattering of radiation. Besides, the third term is corresponded to the gain of energy due to in-scattering of radiation. The parameters *κ*_*λ*_ and *σ*_*λ*_ are the wavelength dependent absorption and scattering coefficients of the medium and *p*(*Ω*′ → *Ω*) is a phase function describing the incident radiation from all other directions surrounding d*s*.

Eqn (10) is used for determination of the incident intensity at any point from all the directions as follows:10
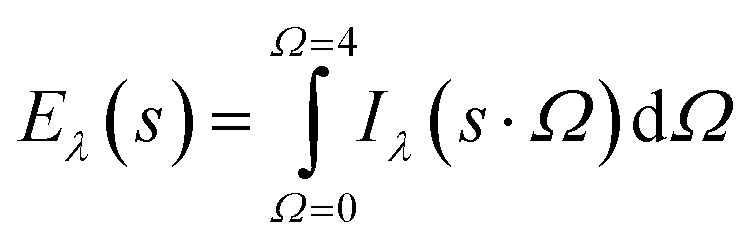


Furthermore, the local volumetric rate of energy absorption (*L*^*a*^) at any point is obtained using following equation:

11 *L*^*a*^_λ_(*s*) = *κ*_*λ*_(*s*)*E*_*λ*_(*s*)

For polychromatic light, radiation is emitted in a range of wavelengths. Consequently, the *L*^*a*^ is summed over the whole absorbable wavelength range. Accordingly, *L*^*a*^ (*s*) is given by eqn (12):12
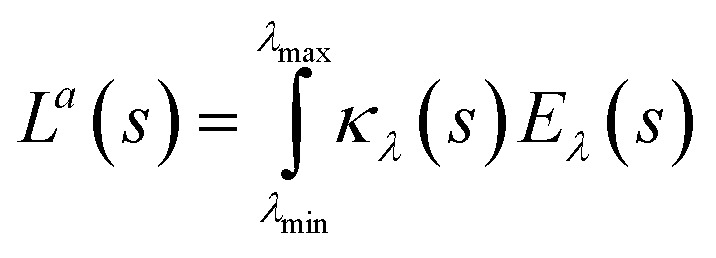


In slurry photocatalytic reactors, the incident radiation changes with separation from the radiation source, as a result, *L*^*a*^ also being varied. In this regard, the volume-averaged *L*^*a*^, 〈*L*^*a*^〉, is used as follows:13
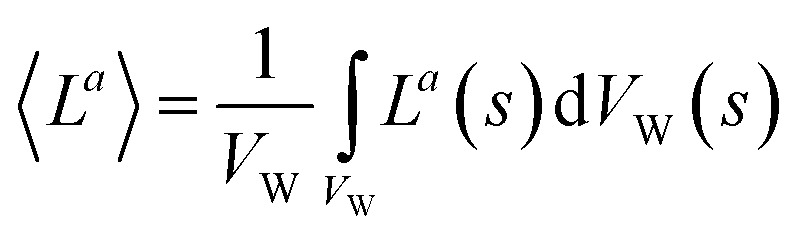
where *V*_W_ is related to the volume occupied by the MP solution.

Furthermore, the overall quantum yield which is defined as the ratio of the volume-averaged rate of reaction to the volume-averaged rate of photons absorbed can be derived *via*eqn (14):14
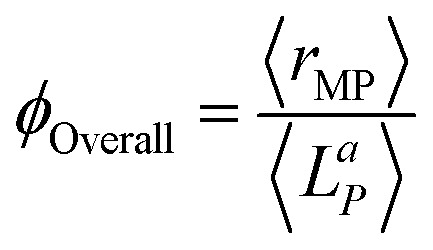
where *r*_MP_ is the MP degradation rate.

For slurry-type photoreactors, owing to light scattering in the presence of the photocatalyst, it is impossible to find an analytical solution for the RTE equation, so a proper mathematical model is required. In this regard, the discrete ordinate method (DOM) was employed based on the finite element method (FEM) to solve the RTE equation (see ESI†). The DOM discretizes the infinite number of directions involved in RTE equation to a finite number of directions, customized to the geometry of the system.

The boundary conditions for solving the above governing equations were considered as follows.

The following boundary conditions are considered for the photocatalytic reactor which their details are identified in Fig. 2c.

Reactor inlet: the initial concentration of MP, and the inlet average flow velocity were specified.

15 *C*_MP_ = *C*^in^_MP_, *u* = *u*_in_

Reactor outlet: at the reactor outlet, an atmospheric pressure boundary condition was applied, and the gradient of species concentration was defined as zero.

16 *P* = *P*_out_, ∇*C*_MP_ = 0

Reactor walls: the reactor’s walls were set as stationary walls with no slip boundary condition on the walls, flux continuity due to mass transport and chemical reaction was considered.

17 *D*_MP_∇*C*_MP_ = *r*′′_MP_, *u* = 0

Reactor surface: a Dirichlet boundary condition was prescribed at the surface for the light intensity, considering it directly exposed to the external photonic flux.

18 *I* = *I*_0_

A finite element technique was employed for discretizing the governing equations including the continuity, the momentum, the mass balance and kinetics equations along with the boundary conditions applied for MP solution flow. The contaminant flow and photodegradation mass transfer rate were modelled with a 3D solver, while the convergence was evaluated by checking the scaled residuals to a criterion of 10^−4^ for the continuity and momentum variables, and 10^−5^ for the species concentrations.

## 3. Results and discussion

### 3.1. Characterization of the photocatalysts

**Fig. 3:**
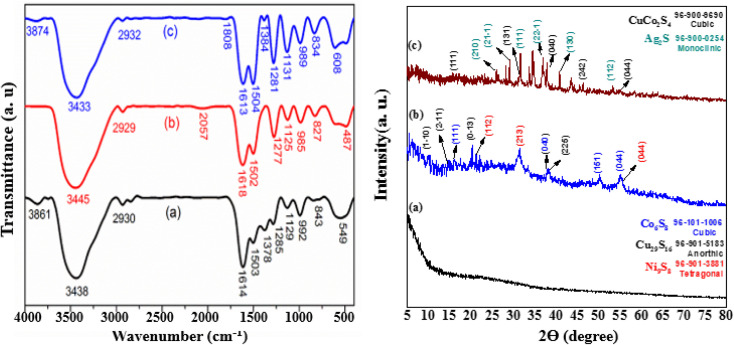
(Left) FTIR spectra of (a) curcumin–zirconium composite, (b) curcumin–zirconium composite on CuCo_2_S_4_ and Ag_2_S compounds, and (c) a sample of a curcumin–zirconium composite on CuCo_2_S_4_ and NiCo_2_S_4_ compounds. (Right): XRD patterns of Zr/curcumin (a), Zr(CUR)/CuCO_2_S_4_–NiCO_2_S_4_ (b), and Zr(CUR)/CuCO_2_S_4_–Ag_2_S (c).

The FTIR spectra of prepared samples is shown in Fig. 3. In spectrum (b), unlike the previous spectrum, no peak observed in the wave number of 1378 cm^−1^ which can be due to the chemical reaction between the CH_3_ groups in curcumin with the compounds of CuCo_2_S_4_ and Ag_2_S. The wide peak at wavelength 487 cm^−1^ is both wider and shifted to a smaller number of waves compared to the peak of 549 cm^−1^ in the previous sample. These changes are due to the peak overlap of zirconium-containing compounds as well as sulfur-containing metal compounds (CuCo_2_S_4_ and Ag_2_S compounds) in this wave number. Another important difference between spectrum (b) and spectrum (a) is the peak in the wave number 2057 cm^−1^, which is related to the tensile vibration of the S–H–C bonds. The presence of this peak proves the creation of a chemical bond between the CuCo_2_S_4_ and Ag_2_S compounds, and curcumin. In fact, the absence of the peak related to the tensile vibration of CH_3_ bonds and instead the appearance of the peak related to the tensile vibration of S–H–C bonds confirms the formation of a chemical bond between the ethyl groups in curcumin and sulfur heteroatoms in CuCo_2_S_4_ and Ag_2_S. In spectrum (c), the decrease in the peak intensity related to the C–H tensile vibration in this sample compared to the sample shown in spectrum (a) and also the emergence of a new peak related to C=O at 1808 cm^−1^ wave number can indicate the participation of these bonds in chemical and physical adsorption of NiCo_2_S_4_ and CuCo_2_S_4_ compounds on the composite. Comparing the intensity of the peak located in the wave number 1378 cm^−1^ in spectrum (a) and the peak located in the wave number of 1384 cm^−1^ in the spectrum (c), it is clear that the peak intensity in the spectrum (c) is much lower, which can be another reason to do the chemical reaction between the C–H groups in curcumin with the compounds CuCo_2_S_4_ and NiCo_2_S_4_. Here, a wide peak located at 608 cm^−1^ wave number is observed which is both wider and shifted to more wave numbers compared to the 549 cm^−1^ peak in the bed sample. This might be due to the peak overlap of zirconium-containing compounds as well as sulfur-containing metal compounds (CuCo_2_S_4_ and NiCo_2_S_4_).

The phase purity of the Zr/curcumin, Zr(CUR)/CuCo_2_S_4_–NiCo_2_S_4_, and Zr(CUR)/CuCo_2_S_4_–Ag_2_S nanocomposites were analyzed using XRD analysis (Fig. 3). The distinct diffraction peaks at 2*θ* of 16.11°, 38.2°, 50.13°, and 55.19° related to the (111), (040), (151), and (044) crystallite planes which is corresponded to the cubic phase of Co_6_S_8_ (JCPDS No: 96-101-1006), while the characteristic peaks of 8.85° (110), 14.72° (211), 21.01° (013), and 37.94 (225) are well adapt to the anorthic phase of Cu_29_S_16_ (JCPDS No: 96-901-5183). Furthermore, the peaks at 2*θ* = 15.13°, 21.53°, 31.11°, 38.17°, and 55.12° can be indexed to the crystallite planes of (301), (112), (213), (004) and (044) corresponded to the tetragonal phase of Ni_9_S_8_ (JCPDS No: 96-901-3881). These evidences confirm the synthesis of Zr(CUR)/CuCo_2_S_4_–NiCo_2_S_4_ composites based on the standard (JCPDS No: 96-900-0254). The diffraction peaks of Ag_2_S at 2*θ* values of 26.38°, 28.99°, 31.59°, 36.9°, 40.75°, and 53.31° were assigned to the reflections (210), (21−1), (111), (22−1), (130), and (112), respectively, which suggested that Ag_2_S possessed the face-centered monoclinic structure. The peaks at 11.22°, 31.47°, 37.92°, and 54.89 are correspond to (111), (131), (040), and (044) crystal phases of CuCo_2_S_4_ (JCPDS file: 96-900-9690) which confirm the synthesis of Zr(CUR)/CuCo_2_S_4_–Ag_2_S nanocomposite.

**Fig. 4:**
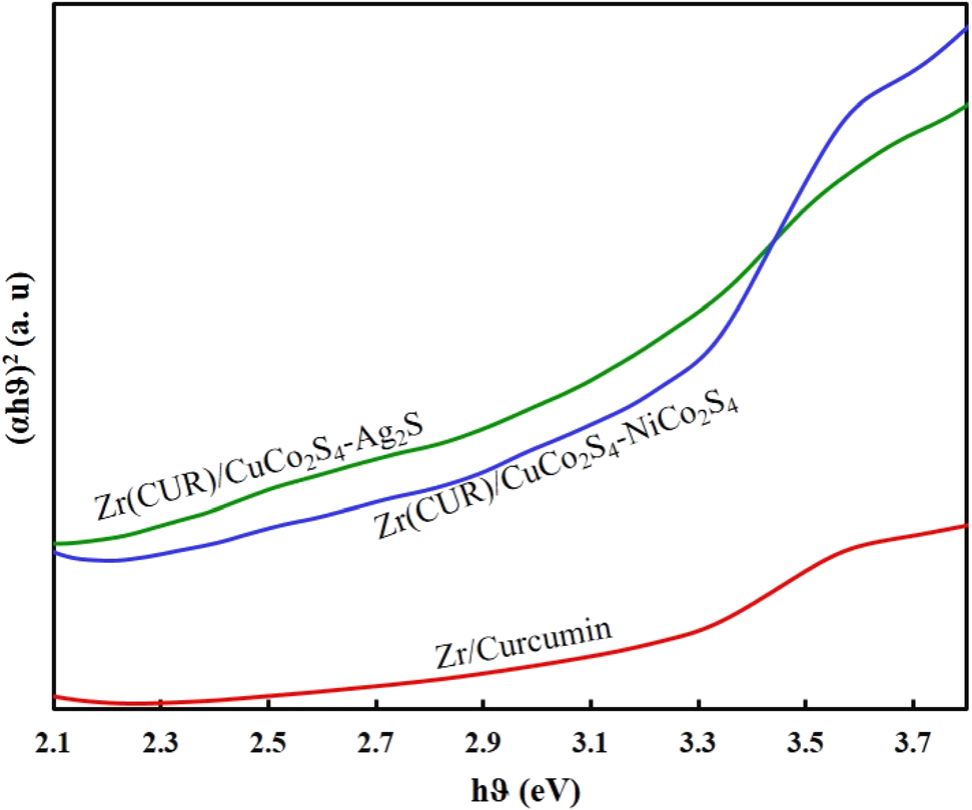
UV–vis DRS patterns of the prepared photocatalyst samples.

The estimated band gaps of prepared samples were obtained employing a Tauc-plot (Fig. 4). As can be seen, all samples can act under visible light irradiation, while the presence of CuCo_2_S_4_, NiCo_2_S_4_, and Ag_2_S can reduce the band gap of Zr/curcumin and improve its photocatalytic activity. Diffuse ref0lectance spectra analysis (Fig. 4) based on well-known equation following plotting (*αhυ*)^2^*versus hυ* and tracing the figures to intercept (*hυ* = 0) indicated that direct band gap was estimated to be 2.81 eV, 2.15 eV, and 2.01 eV for Zr(CUR), Zr(CUR)/NiCo_2_S_4_/CuCo_2_S_4_, and Zr(CUR)/CuCo_2_S_4_/Ag_2_S, respectively.

**Fig. 5:**
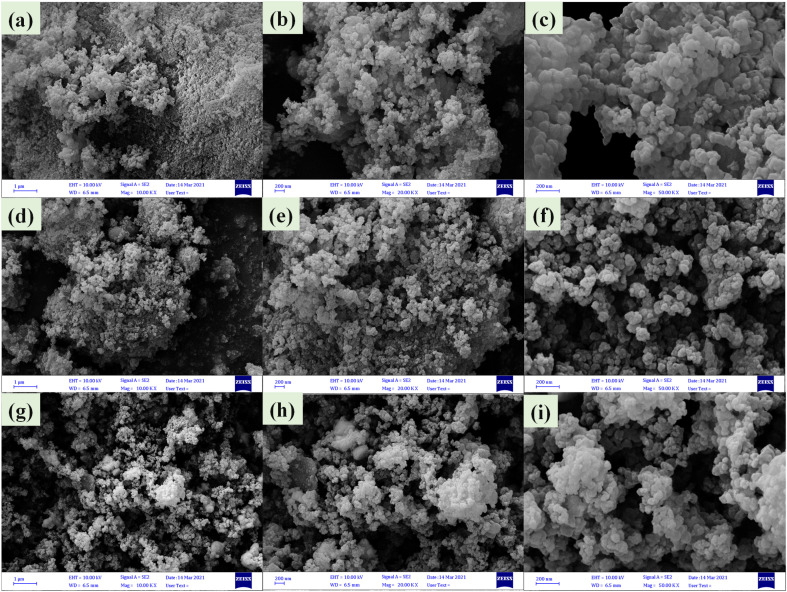
FE-SEM images of Zr/curcumin (a–c), Zr(CUR)/CuCO_2_S_4_–NiCO_2_S_4_ (d–f), and Zr(CUR)/CuCO_2_S_4_–Ag_2_S (g–i).

**Fig. 6:**
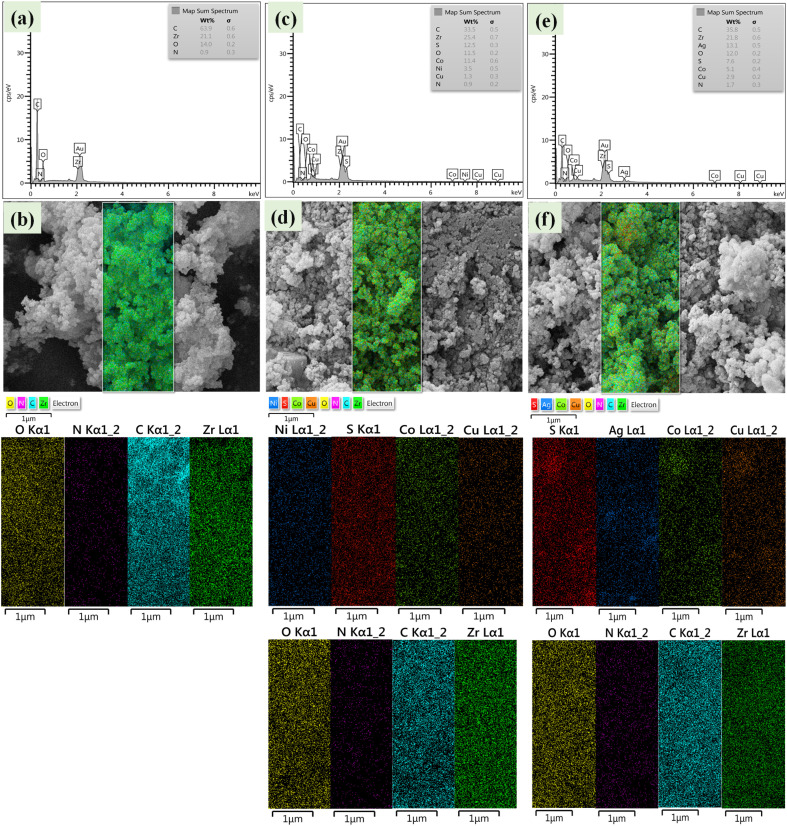
EDS and EDS mapping of Zr/curcumin (a and b), Zr(CUR)/CuCO_2_S_4_–NiCO_2_S_4_ (c and d), and Zr(CUR)/CuCO_2_S_4_–Ag_2_S (e and f).

FE-SEM images of nanoparticles showed that (Fig. 5a–c) Zr/curcumin nanocomposite has an amorphous morphology with a particle size of 60–95 nm. After mixing and homogenizing with CuCo_2_S_4_–NiCo_2_S_4_ (Fig. 5d–f), and CuCo_2_S_4_–Ag_2_S (Fig. 5g–i), no obvious changes were observed in the morphology and the particle size (Fig. 5). EDS and EDS mapping spectroscopy confirmed the presence and dispersion of elements in Zr/curcumin, (CUR)/CuCo_2_S_4_–NiCo_2_S_4_, and Zr (CUR)/CuCo_2_S_4_–Ag_2_S composite which is corresponded to the successful synthesis of these nanomaterials (Fig. 6).

### 3.2. Statistical analysis and CFD results

Flow regime plays an essential role in the degradation of the pollutants using spiral-shaped reactors. The flow pattern in the spiral-shaped photoreactor corresponds to the secondary flow vortices as a result of the Dean flow condition.^40^ Dean flow provides intense mixing conditions, which not only enhances mass transfer rate but also causes further exposure of MP molecules to light in the reactor domain.

**Fig. 7:**
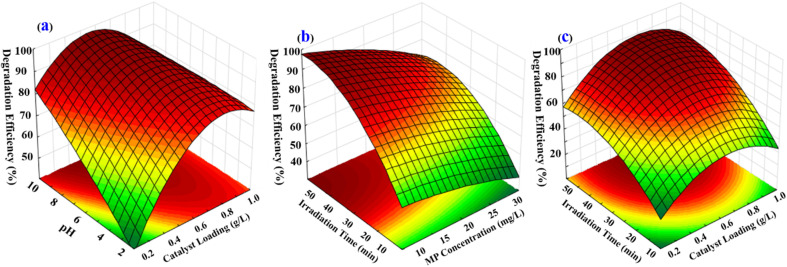
Graphical plots provided by RSM.

The impact of the operational parameters including pH of MP solution, photocatalyst dosage, reaction time and MP concentration on the degradation yield was demonstrated *via* 3D surface plots (Fig. 7). These plots enable the evaluation of the interactions between two operational parameters, while the third parameter is fixed at a certain amount. The interaction between solution pH and the photocatalyst dosage indicated a notable impact on the degradation efficiency of MP (Fig. 7a), while the statistically significant results in Table S4† verify this interaction. The highest MP degradation percentage was related to pH of 8.0. This impact of pH in such value might correspond to the significant electrostatic forces. Findings show that at high MP concentration, the degradation yield is reduced owing to the decline in the available photocatalyst sites (Fig. 7b). The MP degradation yield was intensified as expected by increasing the photocatalyst loading corresponded to more activated sites (Fig. 7c). Furthermore, obtained results revealed that significant improvement of MP degradation was achieved at high reaction time related to the great contact time between MP molecules and photocatalyst particles surface (Fig. 7b and c).

**Fig. 8:**
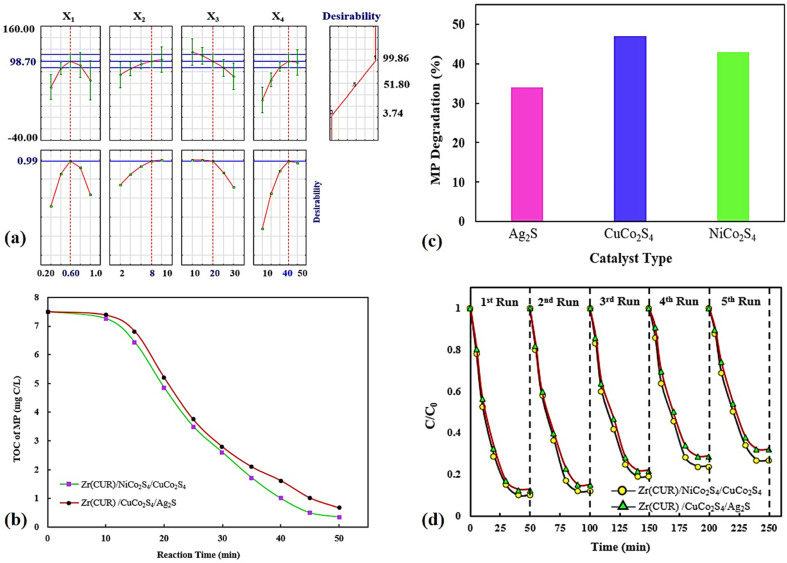
Optimization plots provided by DF (a), TOC tests (b), the contributions of bare samples (c), and valuation of the stability (d).

The desirability function was applied for optimization of the MP degradation process, which findings showed that most MP degradation yield was obtained 98.70% at optimal conditions including 20 mg L^−1^, 0.60 g L^−1^, 8 and 40 min for MP concentration, photocatalyst dosage, pH of solution, and reaction time, respectively with a desirability value of 0.99 (Fig. 8a).

The degradation of the MP pesticide leads to the regular conversion of the present carbon atoms into carbon dioxide and the heteroatoms into different inorganic anions that remain in the solution after the treatment process. The evaluation of TOC to determine the extended mineralization of MP during photocatalysis was carried out at optimum conditions, which findings indicated a considerable reduction in TOC after 50 min. As can be seen in Fig. 8b at the end of treatment, the TOC of the MP was about 91–95% reduced using Zr(CUR)/NiCo_2_S_4_/CuCo_2_S_4_ and Zr(CUR)/CuCo_2_S_4_/Ag_2_S composites, which reveals an improvement in the photocatalytic performance and the oxidation of MP into carbon dioxide.

The effect of bare materials (Fig. 8c) on the MP degradation were investigated which findings showed that degradation percentage obtained using NiCo_2_S_4_ (43.15%) was higher than Ag_2_S (33.87%), but it was lower than to the that of CuCo_2_S_4_ (47.01%).

The durability of the Zr(CUR)/NiCo_2_S_4_/CuCo_2_S_4_ and Zr(CUR)/CuCo_2_S_4_/Ag_2_S composites were evaluated in five successive cycles under identical experimental conditions (Fig. 8d). After each cycle, each photocatalyst was centrifuged, washed and heated at 60 °C. After that, it was applied for the next cycle in the same condition. Findings showed that the both photocatalysts are to be quite stable with superior activity even after five recycles as well as minimal loss in the MP degradation performance.

Adsorption tests were performed to the evaluation of the maximum adsorption capacity (*q*_max_) of prepared samples. Compared with the bare materials, the maximum adsorption capacities of Zr(CUR)/NiCo_2_S_4_/CuCo_2_S_4_ and Zr(CUR)/CuCo_2_S_4_/Ag_2_S composited were obtained 1124.42 mg g^−1^ and 1008.05 mg g^−1^, respectively (Table S5†). It can have concluded that current composites have great potential for the treatment of MP.

The RSM-based equations were derived from quadratic regression fit. Therefore, confirmation tests must be carried out to verify the RSM validity. The independent factors values chosen for the confirmation tests must lie within the ranges for which the formulate were derived. In this regard, five confirmation experiments were performed, while the MP degradation percentage was selected as the indicator. The results of the performed experiments and their comparison with the predict data is listed in Table S6.† As can be seen, the calculated error is small. The error between experimental and predicted values lies within 1.08% to 7.37% which confirms the superior reproducibility of the experimental outcomes.

**Fig. 9:**
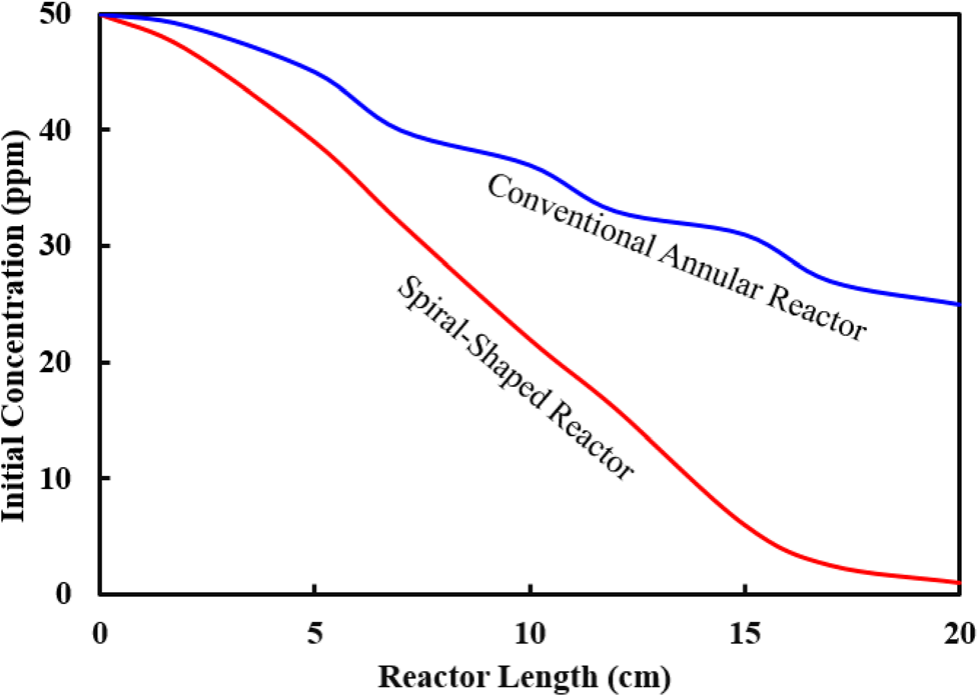
Photocatalytic degradation of MP using different types of reactor geometries.

Finally, the performance of two different photocatalytic reactor geometries (spiral-shaped *versus* traditional annular) was compared for the degradation of MP pesticide, in the same experimental conditions (Fig. 9). Since the target was to compare reactor geometries of similar condition, both the spiral-shaped and the traditional reactors were made using a glass tube with 5 mm inner diameter and 25 cm length. MP degradation yield of 98.70% is obtained with the spiral-shaped reactor, whereas the maximum degradation yield achieved by the annular reactor was 48.62%.

**Fig. 10:**
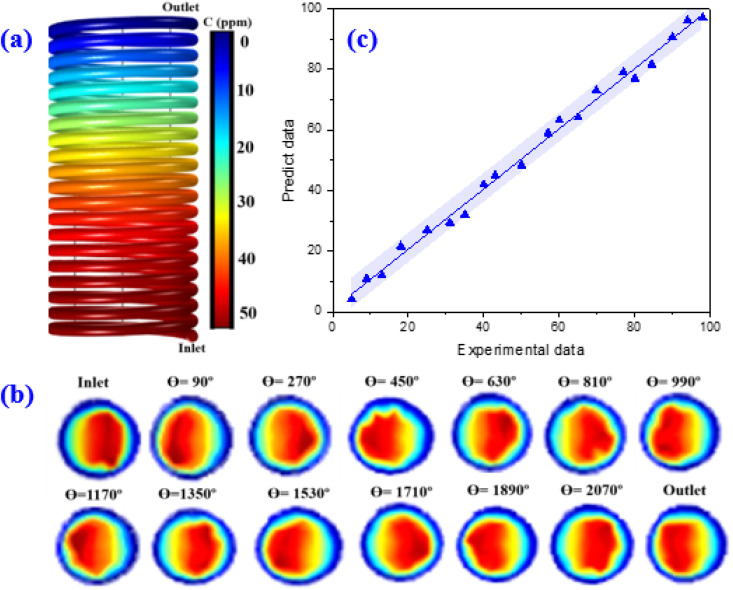
Contour plot of MP concentration during the reactor length (a), the contour plot of velocity magnitude at the different angular plan (b), and the comparison plot of the experimental and predict data for degradation efficiency of MP (c).

The variations of the MP concentrations during the reactor length is shown in Fig. 10a. As can be seen, with the degradation of the contaminants, the outlet concentration of MP from the reactor decreases (Fig. 10a). This high declining rate can be related to the intensification of fluid swirling, which enhances the mass transfer coefficient. Fig. 10b represents the contour of velocity degree ranging with angle and coil turn. The simulation findings indicated that the highest velocity is altered towards the outer wall of the spiral pipe, whereas the velocity starts to alters from angle 90° up to 2070°. As angle is raised, the axial velocity becomes asymmetrical and owing to the unbalanced centrifugal forces on the main flow, the maximum velocity is altered towards the outer wall of the tube.^41^ The findings showed that at a certain angle, as the coil turn increases, the variation in velocity profiles decreases. As can be seen in Fig. 10b, in lower curvature ratios, the mass transfer rate was improved as the helix axial pitch increased. This behaviour could be attributed to the higher surface area achieved in this situation. The flow pattern in the spiral-shape is accompanied by the Dean flow condition, which by the creation of superior mixing conditions, leads to enhancement of mass transfer rate. In fact, under these conditions, solid–liquid interface increases.

The number of coil turns affects the mixing resulting more mass transfer rate. Besides, more exposure to light is provided along the reactor. The spiral reactor not only provides a very homogeneous light distribution over the reactor domain but also leads to creating an intense contact between pollutants and the catalyst particles. This reactor has a very compact design for the effective and fast degradation of MP in a short period as compared to conventional photocatalytic reactor types. Furthermore, the residence time in the spiral photocatalytic reactor is shorter than for the conventional annular reactor. In fact, the development of secondary flows in the spiral photocatalytic reactor enhances the radial mixing while keeping a low axial back-mixing behaviour which causes enhancement of mass transfer and leads to narrower residence time distributions. Table S7† provides some quantitative information as a function of the angle of revolution *θ* and the position in the reactor.

Finally, the CFD model was validated by comparing predictions against experimental data. Finding revealed an acceptable agreement between the proposed COMSOL model and empirical data (Fig. 10c).

### 3.3. Mass transfer studies

The mass transfer studies were performed for both spiral-shaped reactor and the conventional tubular reactor. The mass transfer coefficient as an evaluation indicator was obtained using the eqn (19):19
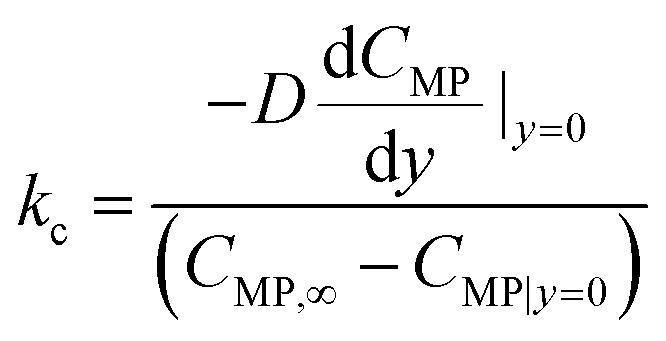
where *C*_MP,∞_ is the MP concentration in the bulk, and *C*_MP|*y*=0_ is the average MP concentration at the catalytic surface. *D* denotes the MP molecular diffusion coefficient in the mixture.

The mass transfer coefficient can be used to calculated the Sherwood number (Sh) as follows:20
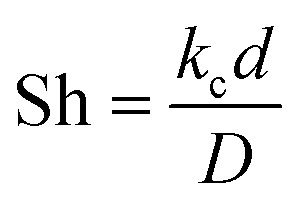
where *d* is the pipe diameter.

**Fig. 11:**
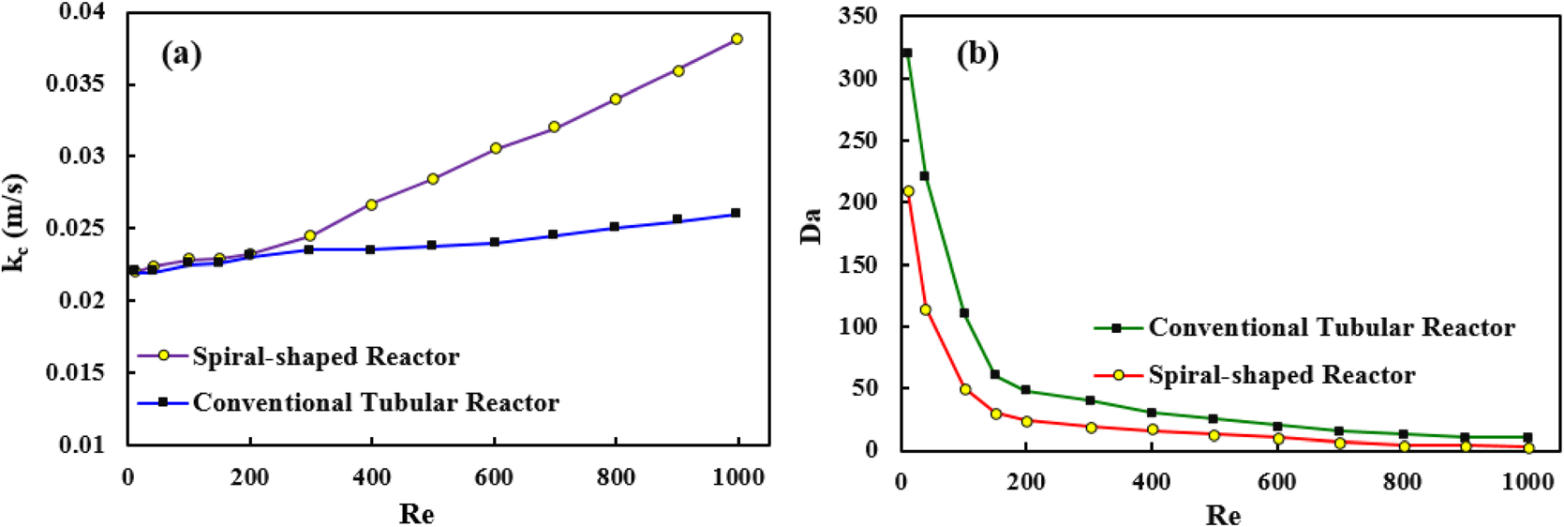
The mass transfer coefficient *versus* Reynolds number (a), and Da number *versus* Reynolds number (b).

The obtained results showed that the spiral-shaped reactor provides higher mass transfer rates when compared to the conventional tubular reactor (Fig. 11a). The enhancement of mass transfer coefficient is corresponded to the more prominent presence of secondary flows in spiral-shaped tubes owing to the centrifugal force induced by the channel curvature. In this condition, the mass transfer occurs not only by the molecular diffusion, but also by the action of the secondary flow in the radial direction, which intensifies the fluid disturbance. Moreover, as can be seen, the mass transfer coefficient increases with an increasing flow rate which means that less resistance exists to mass transfer. Under this situation, the MP outlet concentration decreases significantly. Moreover, the variation of Sh number was obtained based on the eqn (20), which obtained results showed that with the increase of Reynolds number, Sherwood number changes from 6.7 to 12.7. The Sh number increases (less mass transfer resistance) with increasing flow rate which leads to decrease of the MP output concentration and a significant increase of the degradation rate.

Since the dimensionless numbers express the proportion of phenomena in the fluidic parameter space, an additional assessment was performed based on the Damköhler number (Da). The relative importance of the reaction and transport processes in both reactors were evaluated from their respective time scales. Damköhler number was calculated *via* following equation:^42^21
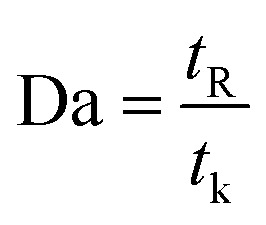
where *t*_R_, and *t*_k_ are the residence time and the reaction time, respectively.

Findings indicated that high values (Da ≫ 1) were observed for both reactor, which revealed that the MP in the reaction medium must have enough time to react (Fig. 11b). Furthermore, the obtained results showed that Da number decreases with increasing Re, which can be explained because the reaction is important for low Re values, while advection becomes the most important process for high values.

## 4. Current challenges and future perspectives

Although the design of the photocatalytic reactors has undergone a rapid progress over the past years, the design, construction, and scale-up of photocatalytic reactors for large-scale industrial applications is still an open problem. Some barriers still need to be overcome. The knowledge of photocatalytic reactor design is a necessary step to appreciating of process performance that involves various aspects of both equipment and process including degradation mechanism used, reactor configuration, hydrodynamic behavior, kinetic model, *etc.* One of the main parameters that should be considered in designing a photocatalytic reactor configuration is the channel length in order to optimize the performance and cost. The increase in the length of reactor increases flow resistance and pumping power. In the straight tubular reactors, the increase in pressure drop was in linear relation with the Re number and length which follows Moody’s chart for friction factor correlation, whereas in the spiral-shape reactors the pumping power increases exponentially with the Re number. However, it should be noted that the spiral-shaped reactor does not involve any moving parts to create the mixing, so unlike reactors that use external energy to induce mixing inside the reactor (using moving parts), a spiral-shaped reactor could lead to energy savings.

In spite of the potential benefits of the photocatalytic degradation process, there are still many problems related to the applicable aspects of photocatalytic reactors. In this respect, the optimal design requires a deep study starting from fluid dynamic considerations, together with the assessment of the light’s distribution inside the reactor domain. A wide variety of reactor configurations have been developed including rotating packed bed reactor, photocatalytic Taylor vortex reactor, fluidized bed reactor, coated fiber optic cable reactor, falling film reactor, thin film fixed bed, swirl flow reactor, and corrugated plate reactor to overcome hindering issues. Future research for designing a reactor for large-scale application should be able to solve several problems such as photon transfer limitation, mass transfer limitation, oxygen deficiency, and lack of reaction pathway control.

## 5. Conclusion

A spiral-shaped photocatalytic reactor was modelled for MP degradation using the CFD technique, while the model verification was performed by comparing the outlet MP concentration with those from the empirical data. The prediction of the velocity distribution and mass transfer rate in the reactor domain were selected as the main goals, while the governing equations consist of the continuity, the momentum, the mass balance and kinetics were solved numerically. Methyl parathion degradation yield of 98.70% is obtained with the spiral-shaped reactor, whereas the maximum degradation efficiency achieved by the annular reactor was 48.62%. Furthermore, the durability of the Zr(CUR)/NiCo_2_S_4_/CuCo_2_S_4_ and Zr(CUR)/CuCo_2_S_4_/Ag_2_S prepared composites were evaluated in five successive cycles under identical experimental conditions which findings showed that the both photocatalysts are to be quite stable with superior activity even after five recycles as well as minimal loss in the MP degradation performance. It is expected that the results of this study could be an essential step during the designing and optimization of the photocatalytic reactors, especially for the scale-up process.

## Conflicts of interest

There are no conflicts to declare.

## Supplementary Material

RA-012-D2RA06277A-s001
