# Supplementary material for: Performance evaluation of Zr(CUR)/NiCo2S4/CuCo2S4 and Zr(CUR)/CuCo2S4/Ag2S composites for photocatalytic degradation of the methyl parathion pesticide using a spiral-shaped photocatalytic reactor
Source: RSC Adv. 2022 Oct 14;12(45):29503–15. doi: 10.1039/d2ra06277a (PMC9562372; doi:10.1039/d2ra06277a)
Supplement: RA-012-D2RA06277A-s001 [file RA-012-D2RA06277A-s001.pdf]

Electronic Supplementary Material (ESI) for RSC Advances.
This journal is © The Royal Society of Chemistry 2022

Performance evaluation of Zr(CUR)/$NiCo_2S_4$/$CuCo_2S_4$ and Zr(CUR) /$CuC_{O2}S_4$/Ag_2S composites for photocatalytic degradation of the methyl parathion pesticide using a spiral-shaped photocatalytic reactor

Hamideh Zolfaghari[a], Fakhri Yousefi[a,*] , Mehrorang Ghaedi[a, **], Soleiman Mosleh[b]

[a] Chemistry Department, Yasouj University, Yasouj 75918-74831, Iran

[b] Polymer Engineering Department, Faculty of Gas and Petroleum, Yasouj University, Gachsaran, 75813-56001, Iran

* Corresponding Authors: fyousefi@yu.ac.ir, *m_ghaedi@yu.ac.ir*

Supplementary information

The details of DOM for solving the radiative transport equation (RTE) in the spiral-shaped photoreactor

The radiative transport equation (RTE) was solved using the discrete ordinate method (DOM) for the description of the light irradiation inside the spiral-shaped photoreactor. The COMSOL Multiphysics® software was used as a powerful CFD tool for numerical simulation. This software has a special Module for solving the radiative transfer equation through discretization. The applied technique contains a numerical integration which considered a set of discrete directions for the radiation and associated quadrature weights. Since the angular space is continuous, the radiative intensity was determined for any direction (Ω), then the specified equations were solved numerically by the discretization of the angular space, as follows [1]:

$$\int_{4\pi} I(\Omega)d\Omega \approx \sum_{j=1}^{n} \omega_j I_j \qquad \text{(S-1)}$$

where in this equation ω_j denotes the i-th quadrature weight.

For any discrete ordinate, Eq. (S-2) was applied as follows [1-3]:

$$S_i \cdot \nabla I_i = \kappa I_b(T) - \beta I_i + \frac{\sigma_s}{4\pi}\sum_{j=1}^{n} \omega_j I_j \Phi\,(S_j.S_i) \qquad \text{(S-2)}$$

where $\mathbf{S}_i$ refers to the *i*-th discrete ordinate, and I_i denotes the *i*-th component of the radiative intensity.

The boundary conditions for solving Eq. (S-2) are presented as follows:

$$I_{i.bnd} = \varepsilon I_b(T) + \frac{1-\varepsilon}{\pi} q_{out} \qquad \text{(S-3)}$$

The q_{out} was calculated using Eq. (S-4):

$$q_{r,out} = \sum_{n \cdot \Omega_j > 0} \omega_j I_j n \Omega_j \qquad \text{(S-4)}$$

The additional computational details are presented at Table S-1, as follows:

Table S1. The additional computational details.

Parameter	Value	Considerations
Photocatalyst absorption coefficient (κ_a)	5.028×10^5 m^{-1}	Estimated at wavelength (λ) of 395 nm
Light intensity	10 $W\cdot m^{-2}$	Imposed on the reactor surface
Unstructured grids	287,000 nodes	1,538,000 tetrahedral cells
Purely absorbent medium	$\kappa_{a=1}$<br>$\sigma_{s=0}$	Used as the boundary condition.
Non-reflective wall	$\varepsilon = 0$	Used as the boundary condition.

[1] https://www.comsol.com/blogs/author/nancy-bannach/ [10/5/2022]
[2]https://www.comsol.com/blogs/heat-transfer-in-participating-media-and-the-discrete-ordinates-method/ [10/5/2022]
[3]https://www.comsol.com/blogs/4-methods-to-account-for-radiation-in-participating-media/ [10/5/2022]

Table S2. Specification of used LED

**Items**	**Min.**	**Type**	**Max.**	**Unit**
Power	-	-	14.40	W/m
Luminous Intensity	1000	-	3500	mcd
Dominant Wavelength	395	-	405	nm
50% Power Angle	-	120	-	Deg.

Table S3. The empirical results of the MP degradation process.

Run	X_1	X_2	X_3	X_4	Y
1	0.4	4	15	20	20.39
2	0.6	6	20	30	77.85
3	0.8	4	15	40	99.86
4	0.6	6	20	30	76.99
5	0.6	6	20	30	77.18
6	0.4	8	15	40	89.53
7	0.8	8	15	20	61.02
8	0.6	6	20	30	77.27
9	0.4	8	25	40	72.01
10	0.8	4	25	40	70.12
11	0.4	4	25	20	3.74
12	0.8	8	25	20	41.50
13	0.6	6	20	30	84.50
14	0.6	6	30	30	57.47
15	0.6	2	20	30	58.34
16	0.6	6	20	30	84.68
17	0.6	6	20	10	19.21
18	1	6	20	30	58.95
19	0.6	10	20	30	98.77
20	0.2	6	20	30	28.84
21	0.6	6	10	30	99.15
22	0.6	6	20	50	98.04

Table S4. The statistical evidence along with analysis of variance.

Source of variation	Sum of Squares	Degree of Freedom	Mean Square	F-Value	P-value
Model	16360.82	14	1168.63	13401.06	< 0.0001
X_1	453.19	1	453.19	5196.91	< 0.0001
X_2	817.29	1	817.29	9372.15	< 0.0001
X_3	1738.68	1	1738.68	19938.02	< 0.0001
X_4	3107.08	1	3107.08	35629.93	< 0.0001
X_1X_2	139.30	1	139.30	1597.39	< 0.0001
X_1X_3	28.46	1	28.46	326.40	< 0.0001
X_1X_4	7.44	1	7.44	85.31	< 0.0001
X_2X_3	10.93	1	10.93	125.31	< 0.0001
X_2X_4	44.28	1	44.28	507.78	< 0.0001
X_3X_4	15.37	1	15.37	176.29	< 0.0001
X_1^2	2743.19	1	2743.19	31457.06	< 0.0001
X_2^2	63.64	1	63.64	729.80	< 0.0001
X_3^2	68.75	1	68.75	788.35	< 0.0001
X_4^2	1122.81	1	1122.81	12875.65	< 0.0001
Residual	0.52	6	0.09		
Lack of Fit	0.095	2	0.05	0.44	0.6694
Pure Error	0.43	4	0.11		
Cor Total	16489.14	21			

Table S5. The maximum adsorption capacity (q_{max}) of prepared samples.

**Sample**	**q_{max} (mg. g^{-1})**
Zr(CUR)/$NiCo_2S_4$/$CuCo_2S_4$	1124.42
Zr(CUR) /$CuCo_2S_4$/Ag_2S	1008.05
$NiCo_2S_4$	642.25
$CuCo_2S_4$	595.60
Ag_2S	287.15

Table S6. Confirmation Tests.

Run	X_1	X_2	X_3	X_4	MP Degradation (%)		
					Actual	Predicted	Error (%)
1	0.5	4	20	25	27.02	29.17	7.37
2	0.4	6	15	40	45.28	43.83	3.31
3	0.8	4	25	30	60.45	61.10	1.06
4	0.2	6	10	10	16.29	15.22	7.03
5	0.6	10	30	40	95.33	96.37	1.08

Table S7. The quantitative information for MP degradation along the reactor.

Turn		1		2		3
**°θ**	90	270	450	630	810	990
**MP% Degradation**	3.52	9.25	11.25	24.68	37.99	46.02

Turn		4		5		6
**°θ**	1170	1350	1530	1710	1890	2070
**MP% Degradation**	51.43	60.95	71.01	77.2	84.95	98.78
